# Development of a Flow-through Cell for Ultrasonic Extraction (UE)—Single Particle (SP)—ICP-MS—an Approach for Nano/Microparticle Elemental and Isotopic Analysis

**DOI:** 10.1021/acs.analchem.6c03259

**Published:** 2026-08-05

**Authors:** Molly K. Paul, Sarah E. Szakas, Cyril V. Thompson, Jordan S. Stanberry, Spencer M. Scott, Rachel A. Bergin, Brian W. Ticknor, Hunter B. Andrews, Benjamin T. Manard

**Affiliations:** † Chemical Sciences Division, 6146 Oak Ridge National Laboratory, 1 Bethel Valley Rd, Oak Ridge, Tennessee 37830, United States; ‡ Savannah River National Laboratory, Savannah River Site, Aiken, South Carolina 29808, United States; § Radioisotope Science and Technology Division, 6146 Oak Ridge National Laboratory, 1 Bethel Valley Rd, Oak Ridge, Tennessee 37830, United States

## Abstract

-Nanotechnology is a salient part of the scientific landscape, and analytical approaches are rapidly evolving to enable small-scale characterization of nano- and microparticle compositions and impurities. Here, a flow-through sonicating cell was developed for direct particle extraction from a silicon wafer and integrated with an inductively coupled plasma–mass spectrometer (ICP-MS) for subsequent elemental and isotopic characterization of the released particles. Ultrasonic extraction (UE)—single particle (SP)—ICP-MS offers controlled particle mobilization from solid substrates and allows for increased sample throughput by eliminating the need for pre-extraction of particles and decreasing sample preparation steps. Coupling this device to an ICP-MS with a time-of-flight (TOF) mass analyzer, it is possible to distinguish unique isotopic compositions of the particles. Both tungsten and nickel isotopically tagged particles, which were deposited on Si wafers, are presented here with analysis via UE—SP—ICP-MS. This approach could support efforts in the fields of particle synthesis, nuclear safeguards and forensics, environmental monitoring, and semiconductor industries in which the detection of particles from substrates and wafers is critical.

## Graphical abstract

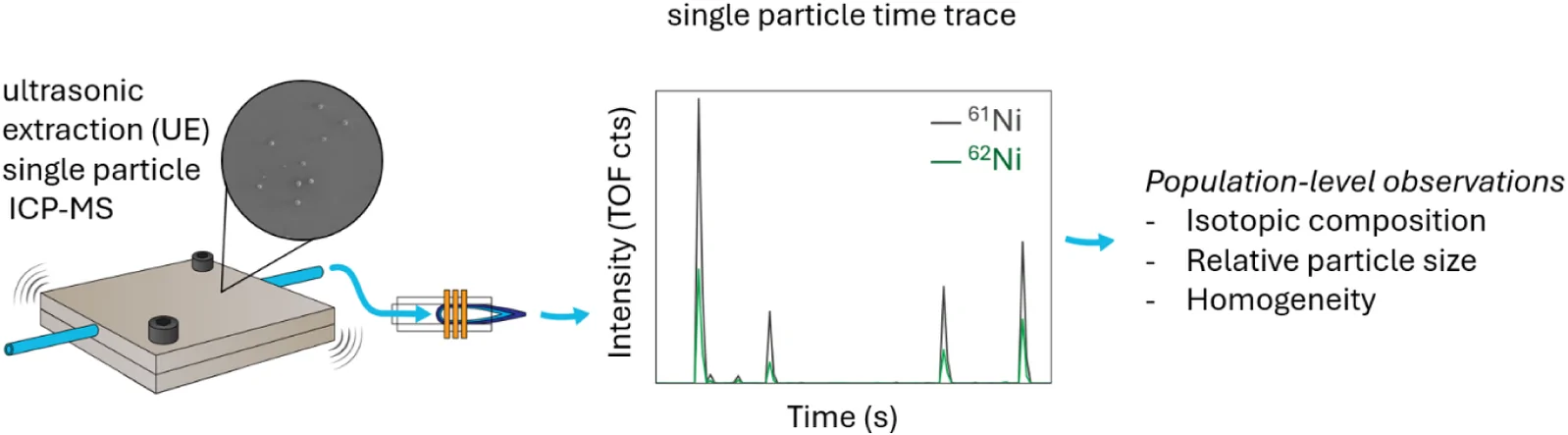

## Introduction

Particles – specifically nanoparticles, microparticles, and colloids – have become an increasingly prominent part of analytical chemistry, with applications across diverse industries. Particles are present naturally as both biogenic (e.g., algae, fungi, proteins) and abiotic (e.g., dust, clay, wildfire aerosols) materials. Anthropogenic sources of environmental particles are often studied in forensics, and can include industrial emissions,^1^ gunshot residue,^2^ and even vehicle brakes.^3^ The industrial applications of particle research are just as broad. Nanoparticles can be used as vaccine platforms,^4^ in biocompatible polymers,^5^ and for degrading environmental pollutants.^6^

Routine particle-characterization methods such as scanning electron microscopy-energy dispersive X-ray spectroscopy (SEM-EDS)^7^ and dynamic light scattering (DLS)^8^ are useful for imaging, elemental identification, and particle-size estimation. However, these approaches are generally low-throughput and offer limited sensitivity, particularly for elemental analysis by EDS. DLS is effective for sizing particles in samples with high particle number concentrations, while SEM-EDS – whether used alone or with automated particle analysis (APA) – supports elemental characterization but cannot scale well for high sample throughput. Importantly, neither SEM-EDS nor DLS can robustly quantify elemental or isotopic compositions at the single-particle or aggregate level.

Another approach is to dissolve the particles into solution. This method is resource-intensive and can obscure particle-level chemical variation; bulk measurements made via inductively coupled plasma–mass spectrometry (ICP-MS) collapse mixed populations into weighted averages, masking heterogeneity. This weighted average result is particularly challenging for nuclear and environmental forensics, where single particle heterogeneity is critical to assessing provenance. To meet the increasing demand for characterization of particles (i.e., elemental and isotopic composition, size, and number concentration), it is necessary to develop robust, cost-effective, high-throughput introduction systems for quantification via ICP-MS.

Single-particle (SP) ICP-MS has become a prominent technique for the determination of particle elemental compositions.^9,10^ SP—ICP-MS differs from conventional, solution-based measurements because signals are discrete rather than continuous. Measuring these discrete signals requires specialized tuning of sample introduction and detector dwell time. This technique can be coupled to a variety of mass analyzers, including quadrupole (Q),^11,12^ multicollector (MC),^13,14^ or time-of-flight (TOF).^15,16^ The quadrupole mass spectrometer is efficient at measurements of a single nuclide, while the multicollector and time-of-flight mass spectrometers specialize in measuring multiple isotopes or elements simultaneously.

Although much of the interest in SP—ICP-MS has been in the past decade, the field has a documented history of innovation. Some of the earliest studies measured Th and U particles using ICP-MS,^17,18^ and established the correlations of particle size to signal intensity and particle number concentration to signal frequency. These developments laid the foundational theory for particle counting and sizing.^19^ However, translating these advancements from suspensions to particles deposited on a substrate remains limited.

Removal of particles from the substrates onto which they are deposited can be a delicate and time-intensive process, and there have been few studies which demonstrate efficient recovery. Often, particles are removed by submerging the deposition medium, usually Si wafers or C planchets,^20^ in water or ethanol and sonicating.^21–23^ While this method does liberate particles, it is tedious, produces dilute suspensions, and particles readily settle if not agitated frequently. Nearly all SP—ICP-MS development has been tailored to suspensions, despite the clear relevance of direct sampling for materials synthesis, semiconductors, and nuclear forensics applications and precedence of particle removal techniques used in nanofabrication (e.g., nanobubble technology,^24^ aerosol mobilization,^25^ laser cleaning^26^). This outpacing of need from method has, at times, produced convoluted sample introduction strategies, and, worse, loss of key population information.

To address this discrepancy, both laser-ablation sample introduction systems^27–29^ and on-surface microextraction devices^30^ have been pioneered. These approaches, combined with sample introduction advancements, have broadened the landscape for SP—ICP-MS. Yet a practical bottleneck remains for routine SP—ICP-MS: efficient mobilization of particles from deposition media into an ICP-MS with high sample throughput.

Described herein is a newly developed sonicating cell which mobilizes particles from Si wafers and directs them into the ICP-MS, ultrasonic extraction (UE)—SP—ICP-MS. Particles on a Si wafer surface are liberated via manually controlled *in situ* sonication and transported into the nebulizer via an isocratic pump with adjustable flow rates. The use of UE—SP—ICP-MS couples sonication of particles on a planchet directly in-line to an ICP-MS sample introduction, allowing for direct and rapid sampling off solid substrates with no need for sample preparation or concern of particles settling. Additionally, this sampling approach is particularly advantageous for situations where particles are likely to degrade after long periods in water or other solvents.

This device was optimized on two ICP-MS platforms (Q and TOF) using an Au nanoparticle (100 nm) standard. This in-line introduction system was further tested using synthesized, isotopically tagged W and Ni microparticles, provided by Savannah River National Laboratory in the form of particles deposited on Si wafers.^31^ Analysis of particles that are fabricated in this way are especially important to the nuclear safeguards community,^32^ which requires well-cataloged particle standards, and this format offers an efficient way to collect and dispatch those standards. Although there are several approaches to studying particles via ICP-MS, this method provides high extraction efficiency and allows control over the proportion of particles entering the MS while maintaining straightforward sample handling. The UE—SP—ICP-MS technique can resolve distinct isotopic populations within a single sample. This capability could support source attribution in nuclear safeguards and forensics, as well as process monitoring for particle analysis such as impurity analysis, and characterization of QC particles.

## Experimental Section

### Materials

#### Au Reference Material Preparation

Au nanosphere suspensions were prepared using gravimetric serial dilutions in water (18.2 MΩ cm). Type I water was generated using a Thermo Scientific Barnstead GenPure xCAD Plus ultrapure water purification system (Waltham, MA). Au nanospheres of 100 nm (102.2 ± 4.2 nm; certificate PNC = 4.9 × 10^9^ particles mL^–1^) were sourced from nanoComposix (San Diego, CA). The final concentration of this solution was 500 particles μL^–1^ and ∼10,000 particles were loaded onto each Si wafer and evaporated^1^ at room temperature until dry.

#### W and Ni Particle Synthesis

Detailed information on feedstock preparation using a double spike method for W (W-α), Ni-α, and Ni-β feedstocks is provided in Bergin et al. 2025 and the supplement, with consensus values for the isotopic composition of each feedstock as determined by independent measurement by two U.S. National Laboratories on a per-mille basis.^31^

In short, dried powders of the prepared feedstocks were dissolved and introduced to the Thermally Evaporated Spray for Engineered Particulates (THESEUS) production platform^33,34^ to produce engineered microparticles with a targeted 1.0 μm diameter. The particles were deposited electrostatically into a circular region 9 mm in diameter onto 1-in. diameter silicon planchets (UniversityWafer, Boston, MA). W and Ni-α particles had a target total particle count of ∼100,000 (5 min collection time), while Ni-β had a targeted particle count of ∼50,000 (3 min collection time). The W particles are estimated to be approximately twice as dense as the Ni particles and slightly larger (W ∼1.2 μm and Ni ∼0.8 μm). Their theoretical masses are ∼1.66 pg W per WO_3_ particle and ∼1.13 pg Ni per NiO particle. No modification of the planchets occurred prior to introduction into the sonicating cell.

## Instrumentation

### SEM Characterization

Samples were imaged using a scanning electron microscope (SEM; SU3800, Hitachi). The silicon wafer was mounted on a 50 mm SEM stub using copper tape, taking care to avoid disrupting particles in the center of the wafer. Secondary electron images were obtained using an accelerating voltage of 2 kV in high vacuum mode and a magnification of both 1,000× and 5,000×.

### UE—SP—ICP-MS

The sonicating cell (Figure 1) was fabricated from polyetheretherketone (PEEK). It is comprised of two halves (upper and lower), which form a seal around the sample via an O-ring (Viton, Wilmington, DE). The lower half has a 1-in. diameter voided, which is recessed to hold the Si-wafer, and the other half has small prongs to provide wafer stability. Solvent (water) from the pump is introduced to the cell via a 0.025-in. diameter path and subsequently fills the internal sample volume of 660 μL, and exits the opposite side toward the ICP nebulizer. The cell is mounted on a sonicating device which is a Langevin-type transducer with a frequency of 28 kHz and a power of 60 W. The transducer is housed in a metal enclosure and coupled to a 1/4-in. aluminum plate to which the sample holder is securely fastened with 6–32 screws.

**Figure 1:**
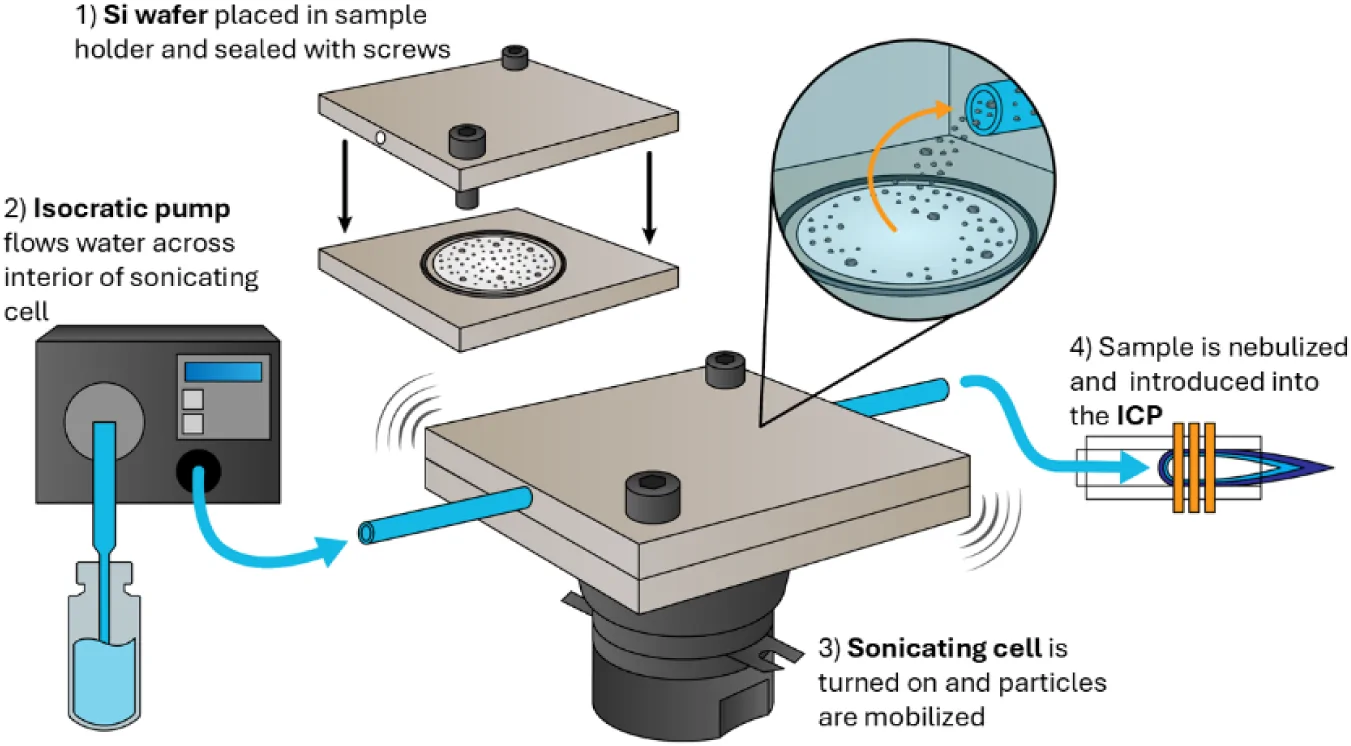
Depiction of the ultrasonic extraction – single particle – inductively coupled plasma–mass spectrometry approach. The sample is placed in the center of the flow through sonicating cell (1), a seal is created within the cell using a rubber O-ring and secured with fasteners. The cell is placed on top of transducer, which can be toggled on and off. An isocratic pump (2) delivers water across the sample surface, the sonication mobilizes the particles (3), and the resulting particles are nebulized and introduced into the ICP (4).

For Au nanoparticle analysis, the sonicating cell was set up in-line with an iCAP Q-ICP-MS (Thermo Scientific, Bremen, Germany). For W and Ni microparticles, the sonicating cell was set up in-line with a TOF R (TOFWERK AG, Thun, Switzerland). To measure larger, and denser, W particles, the TOF was detuned via altering the extraction voltage (voltage reduction from −161.3 V to −57.7 V). With both systems, a PFA MicroFlow nebulizer (Elemental Scientific Inc., Omaha, NE) and Cytospray spray chamber (Elemental Scientific Inc., Omaha, NE) were utilized. Type I 18.2 mΩ cm water was used as the extraction solution. The flow rate through the cell was 100 μL min^–1^, controlled by an isocratic liquid chromatography pump (Advion, Ithaca, NY). Sonication was continuous throughout the analysis duration. The dwell time, or integration time, for data collection from both instruments was 2 ms to ensure single particle events were captured within a few consecutive time bins.

### Measurement Routine

To measure a sonication blank, an empty sonicating cell was placed in-line with the ICP-MS, filled with water, and sonicated. The cell was then disassembled, fully dried with a lint-free laboratory tissue, and the Si wafer was placed inside. The sonicating cell was then filled with water and particles were liberated by activating the sonication.

To preserve the sample postanalysis, sonication was ceased, and the cell was disconnected. The Si wafer was carefully removed from the cell and stored in a 1-in. wafer carrier tray (e.g., Ted Pella, Redding, CA). Any excess liquid within the cell was pipetted into a vial for storage and further analysis. To clean the cell, all components were rinsed in 18.2 mΩ cm water and sonicated twice for a 10 min period. Cells were cleaned with this method after running a blank and sample. As an extra precaution to ensure a completely clean sample introduction, before running the same isotope system again, the components were soaked overnight in trace HCl-HNO_3_. This cleaning procedure ensured no discernible carryover of particles between samples.

### Data Analysis

For Q-ICP-MS analyses of Au standards, particle signals were determined using an iterative thresholding approach.^19,35^ There was no multievent correction applied, which was not problematic for the 100 nm Au particles as typically only one particle was detected within each time bin.

For ICP-TOF-MS, particle signals for W and Ni were determined using TOF-SPI,^36^ a data analysis software specifically used for qualitative and quantitative ICP-TOF-MS data file types. Particle and background signals were determined via compound-Poisson statistics, and critical values (i.e., the empirical count where a particle signal is distinguished from background counts) were established for all isotopes of interest.^37^ Multievent correction was performed by the summation of sequential data points above the critical value, for each isotope individually, into one particle event (SUM-SPECTRUM SE Correction). Output particle intensities were reported as counts. Isotope ratios in each analysis were estimated by calculating the maximum (or maxima) of a kernel density estimation (KDE) of all measured event ratios (eq S1, Figure S1, Figure S2). KDE was performed using in-house Python code built on the SciPy statistics package.

## Results and Discussion

The liberation of particles from solid surfaces via sonication is widely documented.^22,23,38^ Although nanobubbles may also be generated chemically, here nanobubbles are produced when ultrasonic (i.e., high-frequency mechanical acoustic) waves interact with a liquid to produce nanoscale bubbles. When these bubbles implode, they generate microscale jets, shock waves, and shear forces which can disrupt the weak interactions which adhere particles to surfaces.^39^ The main application of this technology has been in cleaning nanoscale components for the semiconductor industry.^40^ The sonicating cell capitalizes on this approach by localizing the sonication to the sample interface and reducing the volume of liquid required. It is hypothesized that the UE approach will be most effective on physically (i.e., weakly electrostatically, in contrast to chemically) adhered particles because they have a larger exposed surface area for nanobubble interaction, although here only physically adhered particles are studied. The sonicating cell was first tested for the UE—SP—ICP-MS analysis of Au nanoparticles deposited on Si wafers to optimize analysis conditions and assess carryover.

### Efficacy of Sonicating Cell ICP-MS

The sonicating cell approach was first validated using Au nanoparticle (100 nm) standards. These standards were gravimetrically prepared, placed in the cell, and sonicated continuously over a 30 min duration while mobilized particles were analyzed via Q-ICP-MS. Several measurement routines were tested, including different flow rates and sonication durations. Flow rates from 10 to 200 μL min^–1^ were tested, as these are commonly used in traditional ICP-MS and single particle methods. Below 100 μL min^–1^, inefficient particle introduction was observed while above 100 μL min^–1^ there were increased occurrences of multiparticle events within single time bins. It was determined that 100 μL min^–1^ was the preferred flow rate to resolve discrete particle signals with efficient sample throughput. This flow rate equates to approximately a 2 min sample uptake time (i.e., duration between beginning sonication and detecting particles). A time trace of the analysis interval (Figure 2) demonstrates the continuous detection of Au particles from the Si wafer. The cumulative particle count over the course of an analysis is plotted in Figure 2. The slope of this line (Figure 2, solid line) indicates that more particles are mobilized during the initial sonication (between 0 and 500 s) than at the end of the analysis duration (>800 s). The moving average particle intensity (window size 100 s) is also shown in Figure 2 (dashed line), which was used to investigate if continuous sonication directly off a surface would induce significant particle degradation or the formation of double events, as indicated by changes to the average particle intensity. The results indicate that the particles were not significantly degraded from extended sonication; the %RSDs of the average signal intensities (i.e., count per second on the quadrupole) from each 100 s window was 11%. Taking the average intensity of all particles detected (λ_Au_ = ∼1.5 × 10^6^ cps), multiparticle events (including double events, etc.) were observed at 8.6% (intensities >2 × λ_Au_).

**Figure 2:**
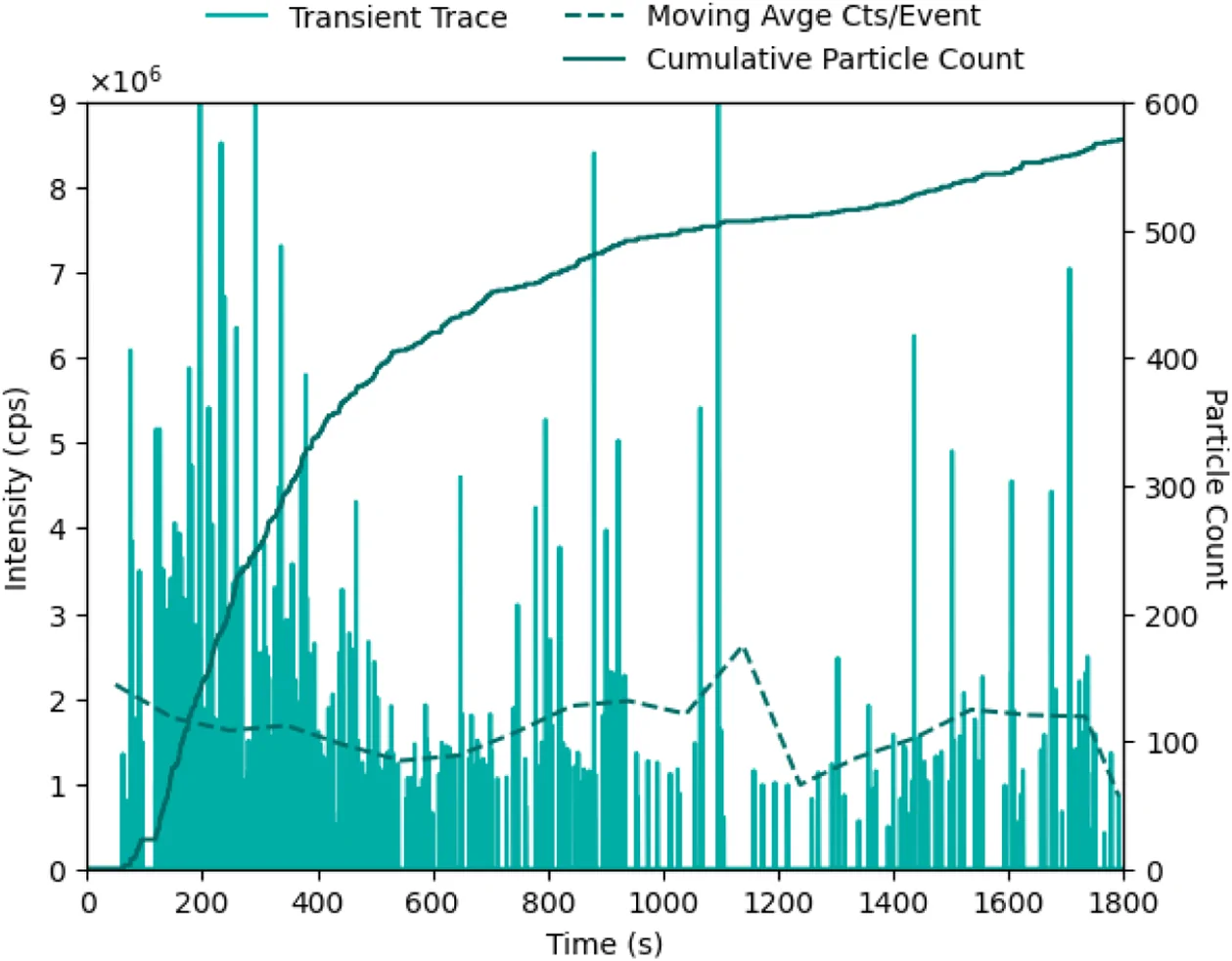
A time trace of signal intensity over a 30 min analysis of Au nanoparticles (100 nm). The dark green solid line follows the cumulative particle count over time (*n* = 571), while the dashed line traces an average particle event intensity every 100 s. Replicate experiments available in Figure S3.

The UE—SP—ICP-MS method yielded an average effective extraction efficiency (eq 1) across triplicate Au experiments of 5.3% ± 0.6% (deposited ∼10K particles wafer^–1^). Effective extraction efficiency is used here to encapsulate particle recovery (removal) by sonication and particle transport (transport efficiency) of ICP-sample introduction. This efficiency could be increased with increased analysis time or different sample introduction systems (e.g., nebulizer and spray chamber). Note that a reasonable estimate of transport efficiency for many nebulizer – spray chamber pairs is about 5%.^41^ This estimate, when compared to the measured extraction efficiency, suggests the primary limitation to higher extraction efficiency is transport efficiency. There are a variety of mechanisms by which transport efficiency can be increased, including direct injection.^42,43^

1 $$extraction\;efficiency=\frac{n\mathrm{o.}\;of\;particles\;detected}{n\mathrm{o.}\;of\;particles\;deposited}\times 100\%$$

### Isotopic Characterization of W Particles via UE—SP—ICP-MS

Of critical importance to particle characterization is the capability to measure isotopic ratios at the single-particle scale. Because W forms semiconducting metal oxides with a range of oxidation states, it is utilized as both a primary constituent and dopant in particles, with applications in gas sensing^44^ and photochromic thin films.^45^ W powders (nano to micro sized) can be utilized for X-ray radiation attenuation in medical applications.^46^ Furthermore, due to its high melting temperature and resistance to corrosion, W is often used to reinforce high performance materials.^47^

W particles on the Si wafers were analyzed via UE—SP—ICP-TOF-MS. Figure 3 shows SEM images of the particles prior to UE—SP—ICP-MS analysis. The resultant time-resolved data from the analysis of W particles is provided in Figure 4 and demonstrates the applicability of the UE—SP—ICP-MS method for isotopic analysis. For each particle event, multiple W isotopes (i.e., ^182^W, ^183^W, ^184^W, and ^186^W) are resolvable (Figure 4). These transient signal spikes are typical in SP—ICP-MS analysis, as individual data points are being integrated every 2 ms, allowing for ionized particle packets which have short-lived temporal durations, to be detected.

**Figure 3:**
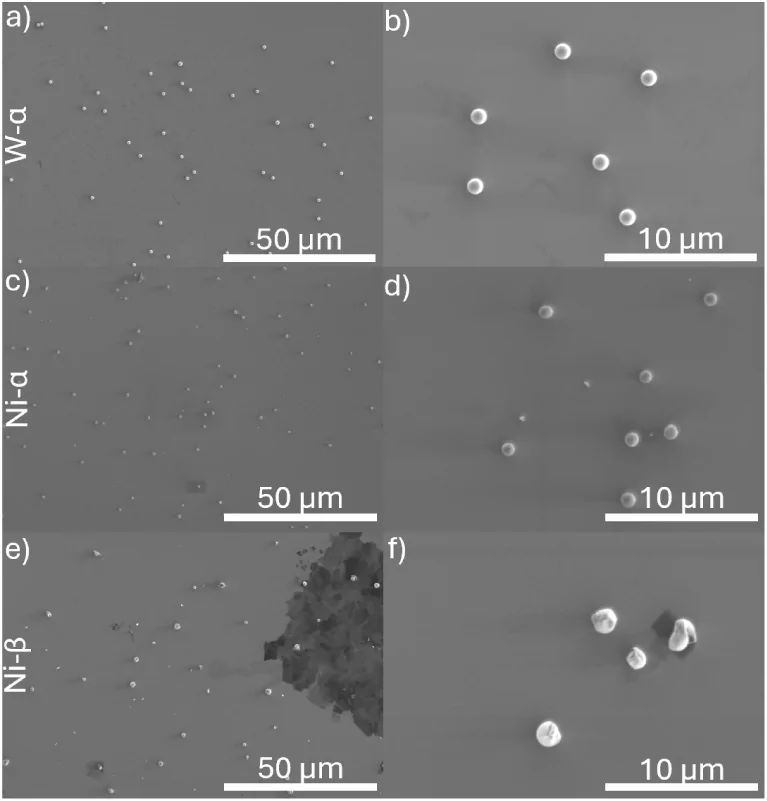
SEM images of particles on Si wafers. Rows correspond to sample (W, Ni-α, and Ni-β), while columns correspond to magnification (1,000× and 5,000×). Complementary postanalysis images of Ni-α are available in Figure S5. (a) is W at 1,000×, (b) is W at 5,000×, (c) is Ni-α at 1,000×, (d) is Ni-α at 5,000×, (e) is Ni-β at 1,000×, and (f) is Ni-β at 5,000×.

**Figure 4:**
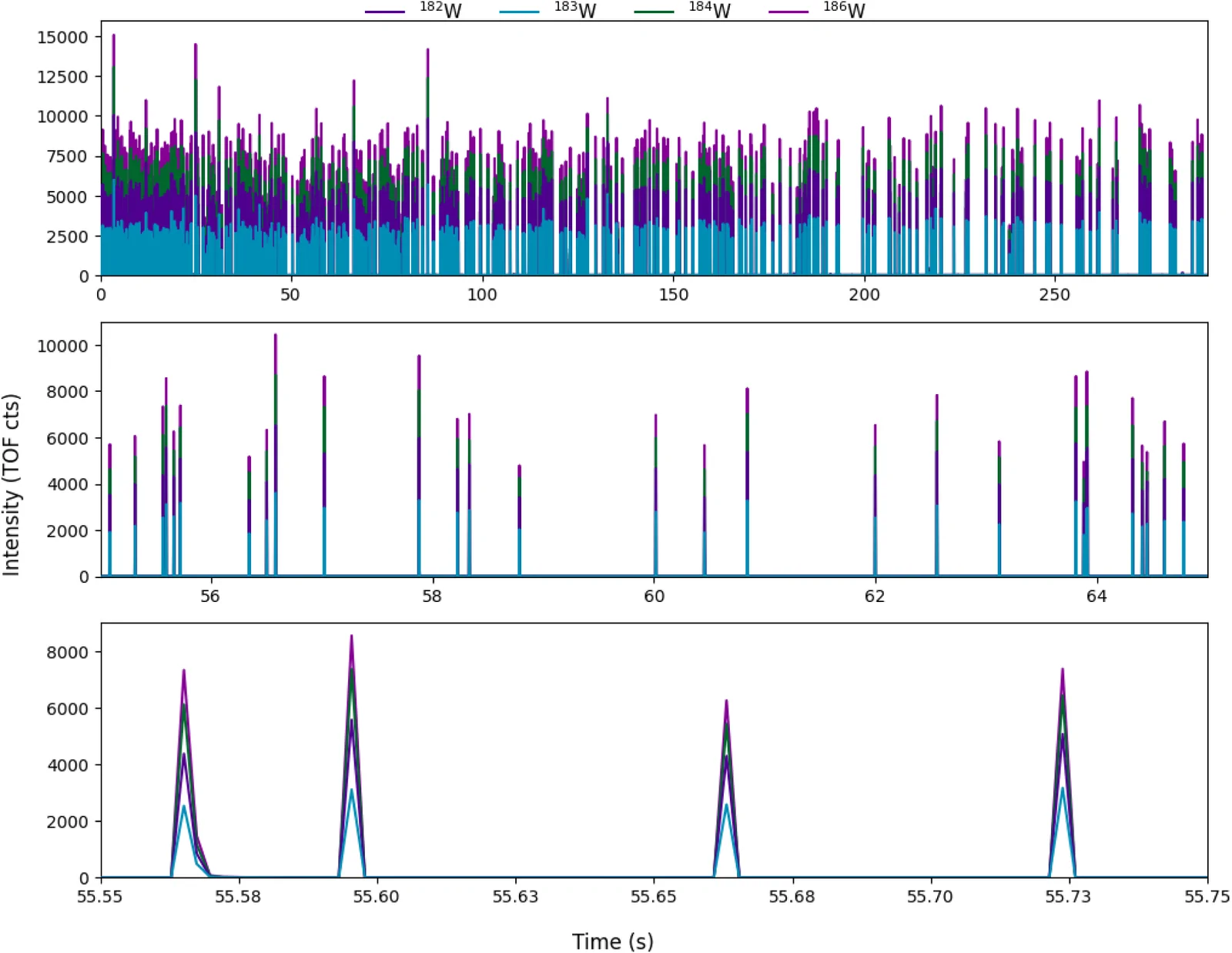
A time trace of the W isotopes (^182^W, ^183^W, ^184^W, and ^186^W) in the W-α particles over a 5 min analysis period. The upper panel depicts the entire analysis duration, while the lower panels depict increasingly smaller time windows.

The W particles, shown to be ∼1 μm in diameter from Figure 3a, b SEM images, are also known to have a density of 4.01 g/cm^3^. Due to the size and density of these particles, initial sample runs showed their signal intensities extended beyond the linear dynamic range of the TOF. To be able to detect all W isotopes of interest (^182^W, ^183^W, ^186^W), the extraction lens of the TOF was detuned. By dropping the extraction voltage by ∼2×, less ions were focused and extracted into the flight tube, effectively decreasing sensitivity of the measurement. To ensure the signals from the main particle population are being used to report isotope ratios, only particles with a minor isotope (^183^W) less than or equal to 2500 TOF counts are considered during data analysis.

The isotope ratio compositions of individual particles are shown in Figure 5 and reported in Table 1. Specifically, ratio analysis was performed on the perturbed ratio (^186^W/^184^W) versus the expected feedstock ratio. From measuring n = 571 particles, the ratio obtained via the maximum of the KDE was 1.172.

**Figure 5:**
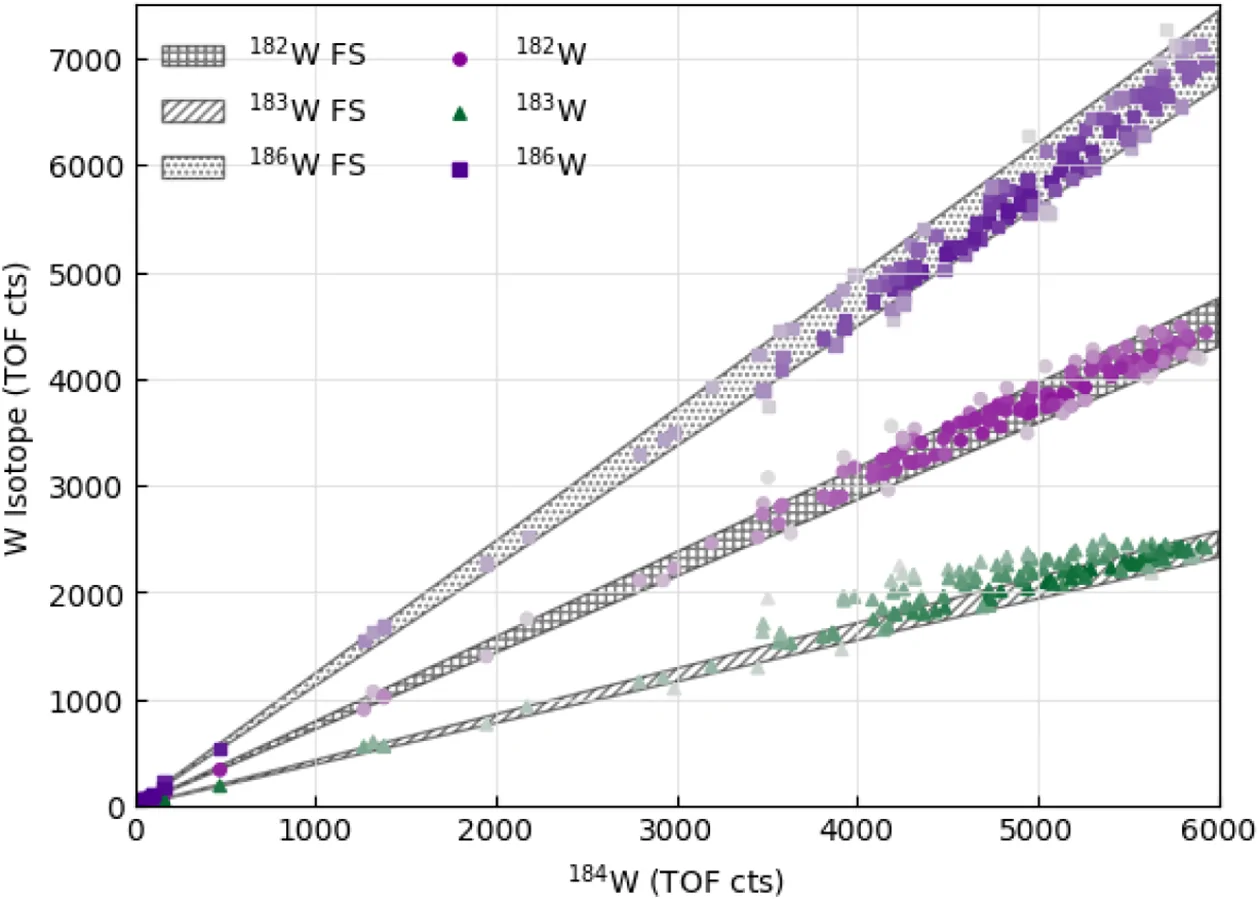
Plot of ^182^W, ^186^W, ^183^W isotope TOF counts versus ^184^W TOF counts. The shaded regions represent ±5% of the measured isotope ratio of the feedstock (FS) from Bergin et al. 2025. All points are colored by a kernel density estimate and range from dark (highest density) to light (lowest density).

**Table 1:** Expected and Estimated Measured Values from Synthesized Particles^a^

	Expected Feedstock Ratio	Measured Feedstock Ratio	Measured Particle Ratio^b^	Percent Relative Difference (Meas Feedstock vs Particle Peak)
W (^186^W/^184^W)	1.226	1.181	1.172	0.8%
Ni-α (^62^Ni/^61^Ni)	0.378	0.388	0.396	2.1%
Ni-β (^62^Ni/^61^Ni)	11.285	10.826	12.265	13.3%

a Expected and measured feedstocks calculated from Tables 2, 4, 5, and 7 in Bergin et al. 2025.^31^

b Measured particle isotope ratios determined by using the maximum of a kernel density estimation of measured particle isotope ratios.

For all isotope ratios in Figure 5, variation in the proportion of reported isotope compositions is visualized using windows within 5% of the measured feedstock ratio (hatched zones). For ^186^W/^184^W, 71.7% of the measured particles are within the measured feedstock ratio. For ^182^W/^184^W and ^183^W/^184^W, the values are 64.1% and 41.0%, respectively. These percentages are correlated with isotopic abundance (R^2^ = 0.987). This discrepancy could be an uncorrected mass bias artifact in this TOF data set, which would likely derive from the detuning of the extraction lens voltage to measure the large, dense W particles by reducing overall sensitivity.^48^ The result of this detuning is that lower mass isotopes have more precisely and accurately measured compositions. Because the extraction voltage was lowered, the extraction efficiency of higher mass ions seems to be altered, and trends toward being less efficient. Further investigation via high precision isotope ratio measurements of the W particles utilizing secondary ion mass spectrometry (SIMS) could provide insights into these differences.

Ultimately, the most isotopically perturbed ratio (^186^W/^184^W), is within 1% of the measured feedstock composition (Table 1), pointing to consistent isotopic modification of the particles during synthesis as well as accurate measurement by the UE—SP—ICP-TOF-MS method. The expected feedstock ratio is slightly higher (∼4%) than both the measured feedstock ratio and the measured particle ratio.

While we see the main population of particles ∼2000 TOF counts of ^184^W in Figure 5, there is a multimodal distribution of particle intensities with a spike of very low count (<100 TOF counts) events. These low count events most likely represent small fragments of particles which either degraded prior to analysis or were fine residues left over from synthesis, comprising <10% of measured particles. These small particles were not readily observed in SEM images (Figure 3). These low count particles, however, do fall within the expected ±5% feedstock (FS), shown by the hatched regions in Figure 5, showcasing not only the use of TOF to accurately determine isotope ratios for the main particle population (1 μm particles) but also its potential use for process monitoring in particle production.

### Ni Particle Characterization via UE—SP—ICP-MS

Synthesized Ni particles, often in the form of Ni oxides or Ni ferrite, or environmentally present Ni-rich materials from both natural and anthropogenic sources, such as soils and fly ash, present another avenue of research interest in Ni isotopic compositions at the single-particle scale. Ni particles are part of a variety of scientific landscapes, including spintronics,^49^ anticancer drug delivery,^50^ and battery electrochemistry.^51^ The isotopic composition of environmental Ni particles can provide insights into biogeochemical cycles, and in some cases trace sources of environmental pollution.^52,53^

Two isotopically perturbed Ni particle populations (Ni-α, Ni-β) were measured via UE—SP—ICP-TOF-MS in order to discriminate between the respective isotopic compositions. α Particle wafers contain ∼100,000 particles, while β particle wafers contain ∼50,000 particles due to the time of collection during production. While isotope ratios for these particle types were originally determined by measuring the altered feedstock composition (Table 1),^31^ it is important to assess the isotope ratios obtained within individual particles to validate the particle-synthesis method. Using a sonication duration of 5 min and a flow rate of 100 μL min^–1^, a sufficient number of particles for isotope characterization (>500 particles) were mobilized. An example time trace of the extracted particles is presented in Figure S4. The isotope ratio distributions derived from particle signals give insights into population characteristics that are otherwise obscured. Isotope compositions are described in terms of ^62^Ni/^61^Ni because these are the most perturbed isotopes from the natural composition (Table 1).

The Ni-α and Ni-β particles have distinctly different isotope compositions (Figure 6). Using the peak of the KDE of isotope ratios, Ni-α had a ^62^Ni/^61^Ni ratio of 0.396 while Ni-β had a ^62^Ni/^61^Ni ratio of 12.265 (Table 1). There has been no confirmation, to date, of individual or bulk particle analysis of these synthesized particles, therefore all isotope ratios obtained are compared to the feedstock solution (both estimated and measured compositions). Subtle variations in this reported isotope ratio could be due to a variety of reasons. Additionally, particle statistics (e.g., imprecision of low-count measurements due to Poisson statistics) will produce an apparent spread in low-counts compositions. Considering the use of a KDE-based approach for isotope ratio estimation, the peak of a KDE will depend on bin size and could be potentially biased by imprecise, low-count measurements. However, due to the presence of high-count particles outside of the 2-sigma threshold (Figure 6), it may be possible there are variations in particle isotope ratios within the group which extend beyond the scale of statistical counting artifacts.

**Figure 6:**
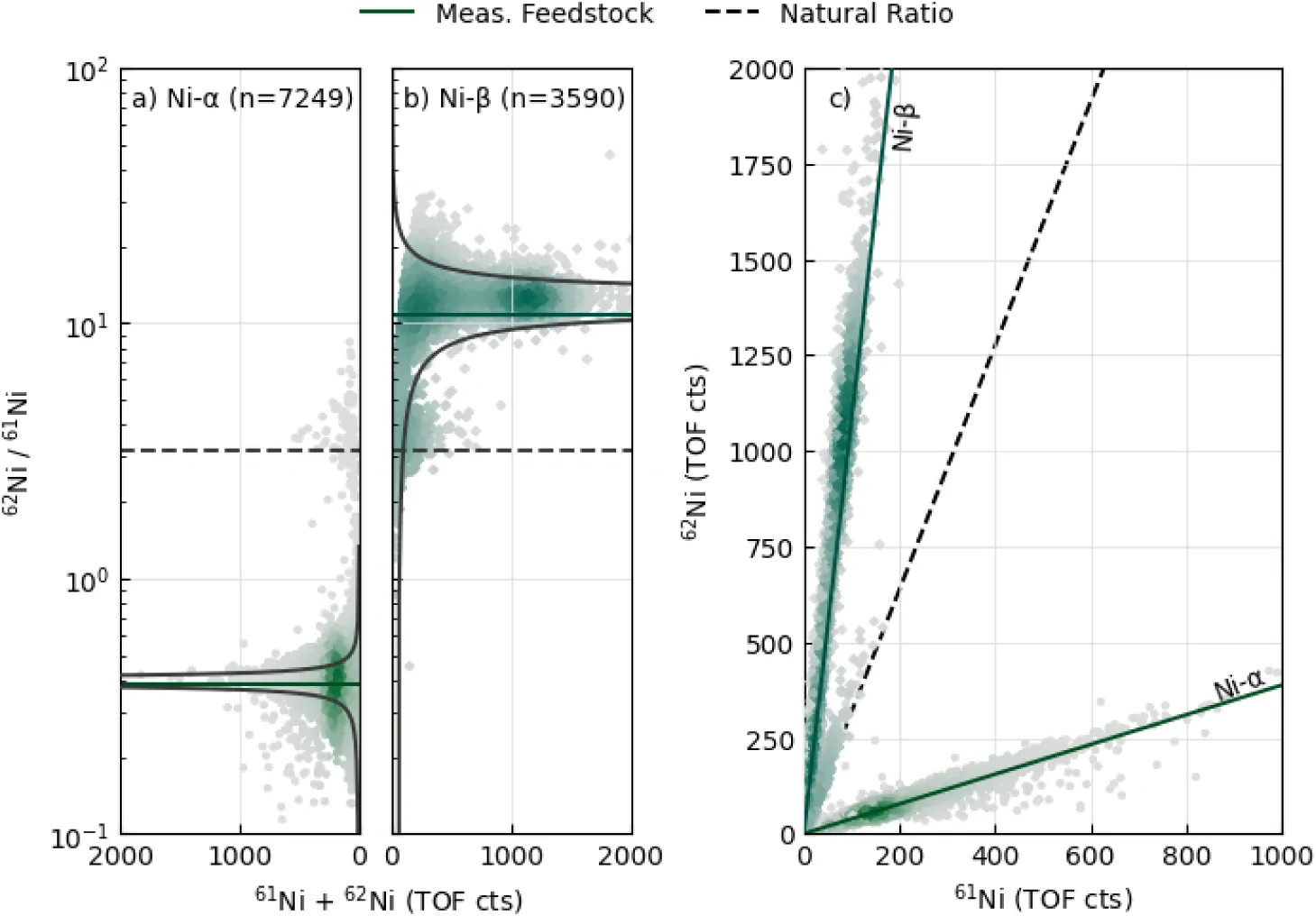
Scatterplots of (a, b) ^62^Ni/^61^Ni over ^61^Ni + ^62^Ni TOF counts and (c) ^62^Ni TOF counts over ^61^Ni TOF counts. Note the inverted *x* axis in plot (b). All points are colored by a kernel density estimate and range from dark (highest density) to light (lowest density). Solid curves in a, b represent 2-sigma Poisson distributions. The measured feedstock ratios are represented with a solid line, while the dashed line represents the natural ratio. The *x* axis of a and b, and both axes of c, are truncated for clarity.

Considering the overall counts of combined ^61^Ni and ^62^Ni in the Ni-α and Ni-β particles, both samples show bimodal distributions (Figure 7). Furthermore, among the densities plotted in Figure 6 (where a dark color indicates a high probability and light gray indicates a low probability), there is also a bimodal distribution of the isotope ratios within the low-count Ni particles in both samples. These low-count particles are related to the sample itself; size variation is observable in SEM images of the Si wafers (Figure 3). Ni-α particles appear to be more homogeneous in shape and size distribution (Figure 3c, d) than Ni-β (Figure 3e, f). SEM images of Ni-α particles postsonication (Figure S5) show no apparent loss of particle integrity postsonication. Interestingly, both Ni-α and Ni-β particles contain a low abundance but measurable group of natural Ni particles (Figure 6). The Ni-β wafer had more natural particles than the Ni-α wafer. The observed nickel particle subpopulation with natural isotopic abundances is hypothesized to be an artifact of particle generation or sample handling.

**Figure 7:**
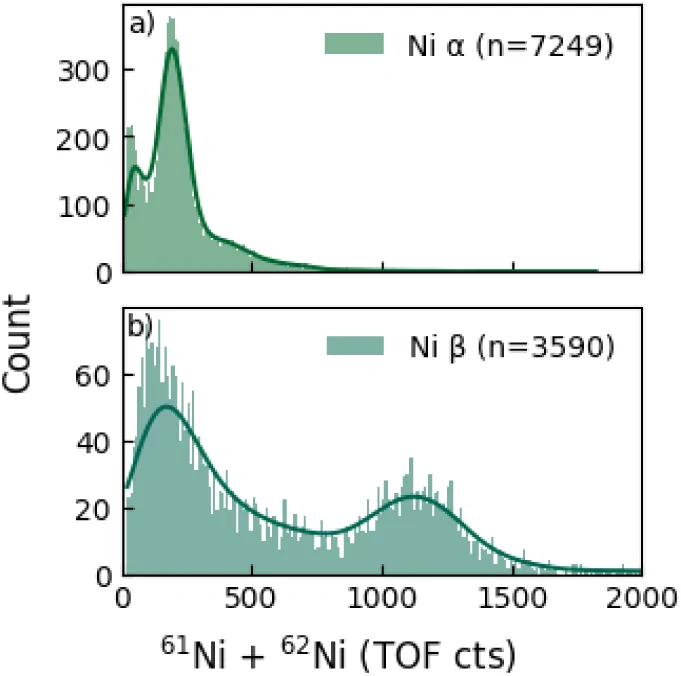
A histogram of relative particle size distribution (proxied by ^61^Ni + ^62^Ni TOF counts) for Ni-α (a) and Ni-β (b). Bin width is 10 TOF counts for both the histogram and kernel density estimate. The *x* axis of (b) is truncated for clarity.

At low counts (<110 ^61^Ni + ^62^Ni TOF counts), isotope ratio uncertainty is too high to reliably distinguish particle populations with similar isotope ratios. About 3.7% of the analyzed Ni-β particles had low enough counts to make them statistically indistinguishable between natural and perturbed Ni. Over 110 ^61^Ni + ^62^Ni counts, particle compositions become resolvable using Poisson counting statistics at a 2-sigma threshold, even for similar isotope ratios (Figure 6). About 62% of measured Ni-β particles were within 2-sigma of the particle feedstock ratio (11.285) and above the count threshold (110 ^61^Ni + ^62^Ni TOF counts) for ratio differentiation. This threshold was determined by calculating the isotope ratio of the lowest-count particle which is within 2-sigma of both the measured perturbed composition and the natural isotope ratio. Because the relative difference of the Ni-α and natural Ni ratio is ∼90%, low-ratio Ni-α measurements were readily distinguishable from natural compositions at 2-sigma. About 60% of measured particles on the Ni-α wafer were within 2-sigma of the particle feedstock ratio (0.378). However, these statistics are likely to represent an underestimation of the total proportion of particles produced from the isotopically perturbed feedstocks measured, as the isotope ratio on a particle-to-particle basis has variation beyond what is accounted for with Poisson counting statistics.

## Conclusions

The principal goal of this research was to demonstrate the capability of a flow through sonicating cell sample introduction system for ultrasonic extraction (UE)—SP—ICP-MS for direct sampling of solid samples for particle analysis. This approach efficiently liberates particles from Si wafers without requiring tedious sample preparation and particle preremoval into a suspension, eliminating sampling bias that can occur at these stages.

Across experiments with 100 nm Au NPs deposited onto the Si wafer, an average extraction efficiency of ∼5.3% was observed. When considering that the typical particle transport efficiency (with the employed flow rate, nebulizer, and spray chamber) is ∼5% it is possible that the UE approach achieves near full recovery of particle removal, and the limiting step is the transport of particles into the ICP. Next, the developed UE introduction was coupled to a simultaneous detection platform, ICP—TOF-MS, which allows for the isotope compositions of individual particles. The integrated approach, UE—SP—ICP-TOF-MS was employed for isotopically characterizing W and Ni isotopes within the respective particles. Briefly, the analysis of W particle was successful at determining the isotopic composition (compared to the bulk starting material used to make the particles such that the ^186^W/^184^W was within 1% relative difference (RD) from the expected value. Regarding the Ni particles, the ^62^Ni/^61^Ni was within 3% and 13% RD for the Ni-α and Ni-β, respectively.

The work presented here truly depicts a novel approach to single particle analysis, allowing for direct mobilization of particles from surfaces. Future research directions will include the following: determination of potential particle size bias derived from sonication-based liberation, quantification of particle mass, assessment of limitations in high-complexity matrices, and applicability to real-world analytical problems. The relevant spaces to apply these advancements include the semiconductor industry, nuclear safeguards and forensics, and environmental monitoring.

## Supplementary Material



## Notes

Experimental details: equations, THESEUS production extended methods, additional KDEs, replicate analyses, additional SEM images, and a table of heating data (PDF)

## References

[1] Goodman A. J., Ahabchane H.-E., Hayes P. L., Wilkinson K. J. (2026). Characterizing
Atmospheric Emissions of Particulate Matter from Copper Smelting through
Coriolis-μ Air Sampling and Single Particle ICP–MS Analysis. ACS EST Air.

[2] Szakas S., Gundlach-Graham A. (2024). Exploring particle populations of
common inorganic
gunshot residue interferences through single particle inductively
coupled plasma time-of-flight mass spectrometry. Talanta.

[3] Grigoratos T., Martini G. (2015). Brake wear particle
emissions: A review. Environ. Sci. Pollut. Res..

[4] Coffman J. E., Metz S. W., Brackbill A., Paul M., Miley M. J., De Simone J., Luft J. C., de Silva A., Tian S. (2018). Optimization
of Surface Display of DENV2 E Protein on a Nanoparticle to Induce
Virus Specific Neutralizing Antibody Responses. Bioconjugate Chem..

[5] Kim J., Pramanick S., Lee D., Park H., Kim W. J. (2015). Polymeric
biomaterials for the delivery of platinum-based anticancer drugs. Biomater. Sci..

[6] Wilcoxon J. P. (2000). Catalytic
Photooxidation of Pentachlorophenol Using Semiconductor Nanoclusters. J. Phys. Chem. B.

[7] Vladár, A. E. ; Hodoroaba, V.-D. Characterization of Nanoparticles by Scanning Electron Microscopy. In Characterization of Nanoparticles; Elsevier: 2020, pp. 7–27.

[8] Ramos, A. P. Dynamic Light Scattering Applied To Nanoparticle Characterization. In Nanocharacterization Techniques; Elsevier, 2017, 99–110.

[9] Montaño M. D., Olesik J. W., Barber A. G., Challis K., Ranville J. F. (2016). Single
Particle ICP-MS: Advances toward routine analysis of nanomaterials. Anal. Bioanal. Chem..

[10] Mozhayeva D., Engelhard C. (2020). A critical review of single particle
inductively coupled
plasma mass spectrometry – A step towards an ideal method for
nanomaterial characterization. J. Anal. At.
Spectrom..

[11] Bolea-Fernandez E., Leite D., Rua-Ibarz A., Balcaen L., Aramendía M., Resano M., Vanhaecke F. (2017). Characterization of SiO_2_ nanoparticles by single particle-inductively coupled plasma-tandem
mass spectrometry (SP-ICP-MS/MS). J. Anal. At.
Spectrom..

[12] Olesik J.
W., Gray P. J. (2012). Considerations
for measurement of individual nanoparticles
or microparticles by ICP-MS: determination of the number of particles
and the analyte mass in each particle. J. Anal.
At. Spectrom.

[13] Manard B.
T., Bradley V. C., Hendriks L., Dunlap D. R., Zirakparvar N. A., Ticknor B. W., Toro-Gonzalez M., Andrews H. B. (2025). Isotopic analysis
of Nd nanoparticles using single particle MC-ICP-MS: A comparative
study with single particle-ICP-TOF-MS. Talanta.

[14] Yamashita S., Yamamoto K., Takahashi H., Hirata T. (2022). Size and isotopic ratio
measurements of individual nanoparticles by a continuous ion-monitoring
method using Faraday detectors equipped on a multi-collector-ICP-mass
spectrometer. J. Anal. At. Spectrom..

[15] Borovinskaya O., Hattendorf B., Tanner M., Gschwind S., Günther D. (2012). A prototype
of a new inductively coupled plasma time-of-flight mass spectrometer
providing temporally resolved, multi-element detection of short signals
generated by single particles and droplets. J. Anal. At. Spectrom..

[16] Ronzani A.-L., Pointurier F., Rittner M., Borovinskaya O., Tanner M., Hubert A., Humbert A.-C., Aupiais J., Dacheux N. (2018). Capabilities of laser ablation – ICP-TOF-MS
coupling for isotopic analysis of individual uranium micrometric particles. J. Anal. At. Spectrom..

[17] Degueldre C., Favarger P. Y., Rossé R., Wold S. (2006). Uranium colloid analysis
by single particle inductively coupled plasma-mass spectrometry. Talanta.

[18] Degueldre C., Favarger P. Y. (2004). Thorium colloid analysis by single
particle inductively
coupled plasma-mass spectrometry. Talanta.

[19] Pace H. E., Rogers N. J., Jarolimek C., Coleman V. A., Higgins C. P., Ranville J. F. (2011). Determining Transport
Efficiency for the Purpose of
Counting and Sizing Nanoparticles via Single Particle Inductively
Coupled Plasma Mass Spectrometry. Anal. Chem..

[20] Richter S., Truyens J., Venchiarutti C., Aregbe Y., Middendorp R., Neumeier S., Kegler P., Klinkenberg M., Zoriy M., Stadelmann G. (2023). Certification of the
First Uranium Oxide micro-particle reference materials for Nuclear
Safety and Security, IRMM-2329P and IRMM-2331P. J. Radioanal. Nucl. Chem..

[21] Fabricius A.-L., Duester L., Meermann B., Ternes T. A. (2014). ICP-MS-based
characterization
of inorganic nanoparticles—sample preparation and off-line
fractionation strategies. Anal. Bioanal. Chem..

[22] Žerdoner T., Vidmar J., Arah B., Zuliani T. (2025). Determination of airborne
metal-containing nanoparticles in a historic mining area using single
particle ICP-MS. Analyst.

[23] Torregrosa D., Grindlay G., De La Guardia M., Gras L., Mora J. (2023). Determination
of metallic nanoparticles in air filters by means single particle
inductively coupled plasma mass spectrometry. Talanta.

[24] Yang S., Duisterwinkel A. (2011). Removal of
Nanoparticles from Plain and Patterned Surfaces
Using Nanobubbles. Langmuir.

[25] Xu K., Pichler S., Wostyn K., Cado G., Springer C., Gale G. W., Dalmer M., Mertens P. W., Bearda T., Gaulhofer E. (2009). Removal of Nano-Particles by Aerosol Spray:
Effect of Droplet Size and Velocity on Cleaning Performance. Solid State Phenom..

[26] Leiderer, P. ; Olapinski, M. ; Mosbacher, M. ; Boneberg, J. Nanoparticle adhesion and removal studied by pulsed laser irradiation; SPIE, 2006.

[27] Metarapi D., Šala M., Vogel-Mikuš K., Šelih V. S., Van Elteren J. T. (2019). Nanoparticle Analysis in Biomaterials Using Laser Ablation–Single
Particle–Inductively Coupled Plasma Mass Spectrometry. Anal. Chem..

[28] Van
Acker T., Rua-Ibarz A., Vanhaecke F., Bolea-Fernandez E. (2023). Laser Ablation for Nondestructive Sampling of Microplastics
in Single-Particle ICP-Mass Spectrometry. Anal.
Chem..

[29] Manard B. T., Szakas S. E., Stanberry J. S., Ticknor B. W., O’Brien L., Boris M., Hewitt J. T., Cable-Dunlap P., Andrews H. B. (2025). Evaluating the feasibility of LA-ICP-TOF-MS for the
analysis of environmental particle collections. J. Anal. At. Spectrom..

[30] Stanberry J. S., Andrews H. B., Thompson C. V., Ticknor B. W., Manard B. T. (2025). Microextraction-Single
Particle-Inductively Coupled Plasma-Mass Spectrometry for the Direct
Analysis of Nanoparticles on Surfaces. Anal.
Chem..

[31] Bergin R., Samperton K., Bronikowski M., Hoar E., Rolison J., Shollenberger Q., Marks N., Wellons M., Scott S. (2025). Synthesis
and characterization of isotopically barcoded nickel, molybdenum,
and tungsten taggants for intentional nuclear forensics. Talanta.

[32] Stanberry J. S., Szakas S. E., Andrews H. B., Hendriks L., Ticknor B. W., Bergin R. A., Tazik S. K., Kegler P., Neumeier S., Zirakparvar N. A. (2026). Rapid isotopic analysis of uranium microparticles
via SP-ICP-TOF-MS. Talanta.

[33] Naes B. E., Scott S., Waldron A., Lawson S., Bronikowski M. G., Gleaton L. I., Smith R. J., Wurth K. N., Tenner T. J., Wellons M. (2023). Production of mixed element actinide reference particulates
to support nuclear safeguards using THESEUS, an aerosol-based particulate
synthetic methodology. Analyst.

[34] Scott, S. M. ; Baldwin, A. T. ; Bronikowski, M. G., II ; De Vore, M. A. ; Inabinet, L. A. ; Kuhne, W. W. ; Naes, B. E. ; Smith, R. J. ; Villa-Aleman, E. ; Tenner, T. J. ; Scale-up And Production Of Uranium-bearing QC Reference Particulates By An Aerosol Synthesis Method. In Joint INMM ESARDA Meeting, Virtual; Institute of Nuclear Materials Management (INMM), 2021.

[35] Andrews H. B., Hendriks L., Irvine S. B., Dunlap D. R., Manard B. T. (2025). TOFHunter—unlocking
rapid untargeted screening of inductively coupled plasma–time-of-flight–mass
spectrometry data. J. Anal. At. Spectrom..

[36] Gundlach-Graham A., Harycki S., Szakas S. E., Taylor T. L., Karkee H., Buckman R. L., Mukta S., Hu R., Lee W. (2024). Introducing
“time-of-flight single particle investigator” (TOF-SPI):
A tool for quantitative spICP-TOFMS data analysis. J. Anal. At. Spectrom..

[37] Gundlach-Graham A., Lancaster R. (2023). Mass-Dependent Critical Value Expressions for Particle
Finding in Single-Particle ICP-TOFMS. Anal.
Chem..

[38] Lu H., Huang F., Guo H. (2021). Differential Removal of Nanoparticles
on the Surface of a Thin Film Substrate. ACS
Omega.

[39] Chahine G. L., Kapahi A., Choi J.-K., Hsiao C.-T. (2016). Modeling
of surface
cleaning by cavitation bubble dynamics and collapse. Ultrason. Sonochem..

[40] Hauptmann M., Frederickx F., Struyf H., Mertens P., Heyns M., De Gendt S., Glorieux C., Brems S. (2013). Enhancement of cavitation
activity and particle removal with pulsed high frequency ultrasound
and supersaturation. Ultrason. Sonochem.

[41] Priede A. S., Corte-Rodríguez M., Gödde H., Bayón M. M., Bettmer J. (2025). Single particle and
single cell analysis
by ICP-MS with pneumatic nebulisation: Comparing sample introduction
systems. Talanta.

[42] Zhou Y., Song X., Yan X., Yang L., Chen S., Wang Q. (2022). Zero-Interfacing μHPLC
to ICPMS. Anal.
Chem..

[43] Zhou Y., Chen Z., Zeng J., Zhang J., Yu D., Zhang B., Yan X., Yang L., Wang Q. (2020). Direct Infusion
ICP-qMS of Lined-up Single-Cell Using an Oil-Free Passive Microfluidic
System. Anal. Chem..

[44] Staerz A., Somacescu S., Epifani M., Kida T., Weimar U., Barsan N. (2020). WO_3_-Based Gas Sensors: Identifying Inherent
Qualities and Understanding the Sensing Mechanism. ACS Sens..

[45] Sun M., Xu N., Cao Y. W., Yao J. N., Wang E. G. (2000). Nanocrystalline
Tungsten Oxide Thin Film: Preparation, Microstructure, and Photochromic
Behavior. J. Mater. Res..

[46] Aral N., Amor Duch M., Banu Nergis F., Candan C. (2021). The effect of tungsten
particle sizes on X-ray attenuation properties. Radiat. Phys. Chem..

[47] Rieth M., Dudarev S. L., De Vicente G. S. M., Aktaa J., Ahlgren T., Antusch S., Armstrong D. E. J., Balden M., Baluc N., Barthe M.-F. (2013). Recent
progress in research on tungsten materials
for nuclear fusion applications in Europe. J.
Nucl. Mater..

[48] Lomax-Vogt M., Carter L. M., Wielinski J., Kutuzov S., Lowry G. V., Sullivan R., Gabrielli P., Olesik J. W. (2025). Challenges in measuring
nanoparticles and microparticles by single particle ICP-QMS and ICP-TOFMS:
size-dependent transport efficiency and limited linear dynamic range. J. Anal. At. Spectrom..

[49] Lüders U., Barthélémy A., Bibes M., Bouzehouane K., Fusil S., Jacquet E., Contour J.-P., Bobo J.-F., Fontcuberta J., Fert A. (2006). NiFe_2_O_4_: A
Versatile Spinel Material Brings New Opportunities for Spintronics. Adv. Mater.

[50] Joshy K. S., Augustine R., Mayeen A., Alex S. M., Hasan A., Thomas S., Chi H. (2020). NiFe_2_O_4_/poly­(ethylene
glycol)/lipid–polymer hybrid nanoparticles for anti-cancer
drug delivery. New J. Chem..

[51] Kumar
Rai A., Tuan Anh L., Park C.-J., Kim J. (2013). Electrochemical study
of NiO nanoparticles electrode for application in rechargeable lithium-ion
batteries. Ceram. Int..

[52] Ratié G., Quantin C., Jouvin D., Calmels D., Ettler V., Sivry Y., Vieira L. C., Ponzevera E., Garnier J. (2016). Nickel isotope fractionation during
laterite Ni ore
smelting and refining: Implications for tracing the sources of Ni
in smelter-affected soils. Appl. Geochem..

[53] Šillerová H., Chrastný V., Vítková M., Francová A., Jehlička J., Gutsch M. R., Kocourková J., Aspholm P. E., Nilsson L. O., Berglen T. F. (2017). Stable
isotope tracing of Ni and Cu pollution in North-East Norway: Potentials
and drawbacks. Environ. Pollut..

