# Supplementary material for: Development of a Flow-through Cell for Ultrasonic Extraction (UE)—Single Particle (SP)—ICP-MS—an Approach for Nano/Microparticle Elemental and Isotopic Analysis
Source: Anal Chem. 2026 Aug 5;98(32):23774–83. doi: 10.1021/acs.analchem.6c03259 (PMC13492043; doi:10.1021/acs.analchem.6c03259)
Supplement: Supplementary file 1 [file ac6c03259_si_001.pdf]

Supporting Information

Development of a flow-through cell for ultrasonic extraction (UE) - single particle (SP) - ICP-MS – an approach for nano/microparticle elemental and isotopic analysis

Molly K. Paul[1], Sarah E. Szakas[1], Cyril V. Thompson[1], Jordan S. Stanberry[1], Spencer M. Scott[2], Rachel A. Bergin[2], Brian W. Ticknor[1], Hunter B. Andrews[3], Benjamin T. Manard[1*]

1. Chemical Sciences Division, Oak Ridge National Laboratory
   1 Bethel Valley Rd, Oak Ridge, TN 37830

2. Savannah River National Laboratory
   Savannah River Site, Aiken, SC 29808

3. Radioisotope Science and Technology Division, Oak Ridge National Laboratory
   1 Bethel Valley Rd, Oak Ridge, TN 37830

*corresponding author: ManardBT@ornl.gov

This manuscript has been authored in part by UT-Battelle, LLC, under contract DE-AC05-00OR22725 with the US Department of Energy (DOE). The US government retains and the publisher, by accepting the article for publication, acknowledges that the US government retains a nonexclusive, paid-up, irrevocable, worldwide license to publish or reproduce the published form of this manuscript, or allow others to do so, for US government purposes. DOE will provide public access to these results of federally sponsored research in accordance with the DOE Public Access Plan (http://energy.gov/downloads/doe-public-access-plan).

Table of Contents

Extended Methods S1. 3

Figure S1. 4

Figure S2. 5

Figure S3. 6

Figure S4. 7

Figure S5. 8

Equation S1 9

Equation S2. 9

Table S1. 9

References 10

Extended Methods S1.

The THESEUS production platform employs a Flow-Focusing Monodisperse Aerosol Generator (FMAG, Model 1520, TSI Inc.) to produce uniform droplets at a frequency of 130 kHz, flow focusing pressure of 2.00 ± 0.05 psi, and a flow rate of 3.0 mL hr^{-1} from the sample solution. This droplet stream is introduced into a filtered air stream at a flow rate of 10.0 ± 0.1 L min^{-1} to remove excess water from the droplets prior to introduction into a diffusion drier (Model 3062, TSI, Inc.) and inline heater (Thermo Fisher Lindberg Blue M Three-Zone Tube Furnace equipped with a 1-inch Inconel 625 tube) to calcine and oxidize particles to an oxide form. The three-zone tube furnace allows for gradual heating of the aerosol, with an adjustable temperature setting at the inlet, center zone, and heater exit. Calcination and oxidation were completed using the following temperature settings:

- W particles: 600 °C for all zones
- Ni-α: inlet 380 °C; center 500 °C; exit 500 °C
- Ni-β: inlet 330 °C; center 450 °C; exit 600 °C

The particles were deposited electrostatically onto 1-inch diameter silicon planchets (UniversityWafer, Boston, MA) using a 9-mm mask within an SRNL-designed mini-aerosol contaminant extractor (Mini-ACE).

Figure S1. Kernel density estimates for the distribution of measured isotope ratios for Ni-α and Ni -β. Bandwidth is calculated using Eq. S1.

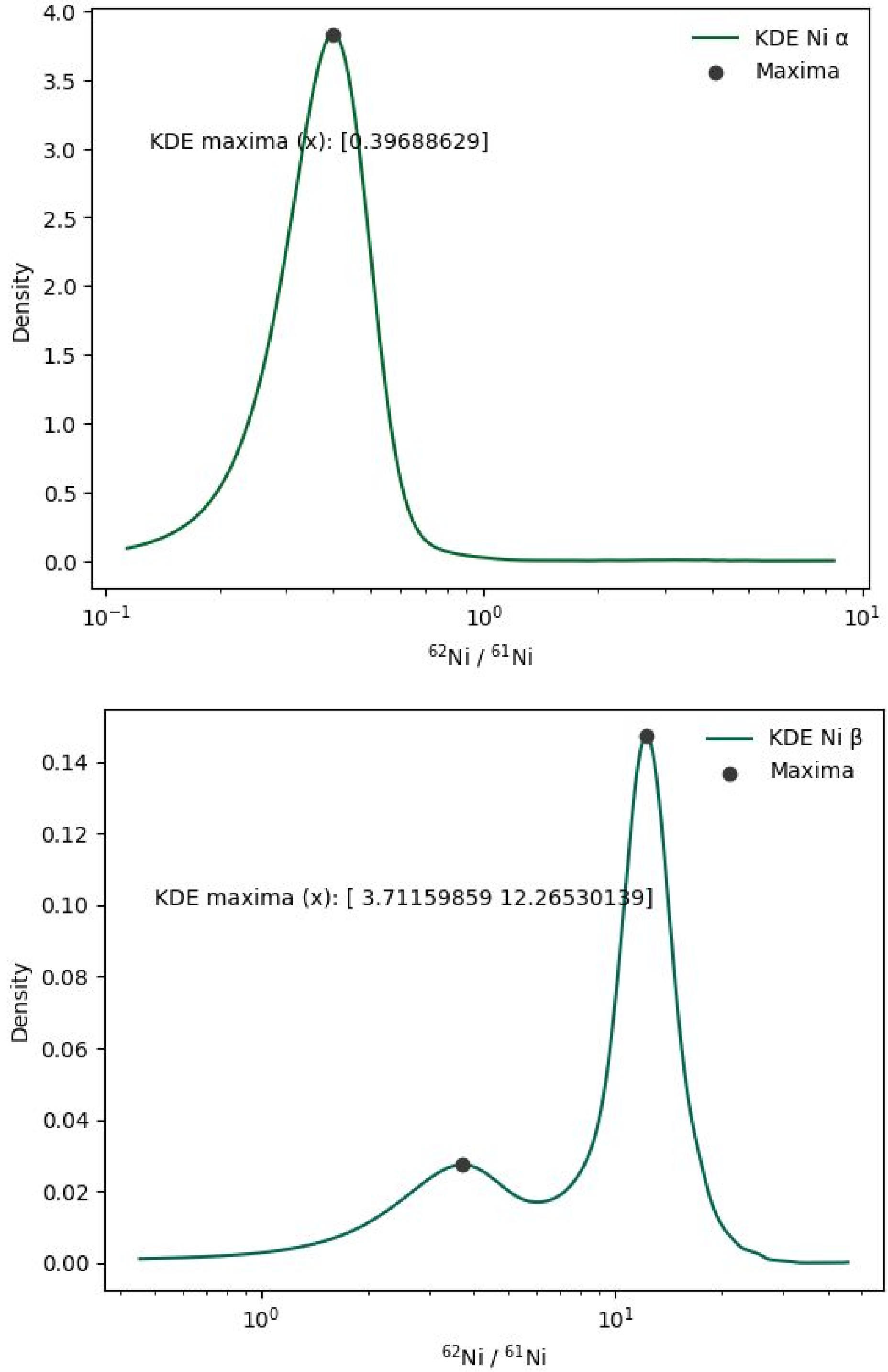

Figure S2. Kernel density estimates for the distribution of measured isotope ratios for W. Bandwidth is calculated using Eq. S1.

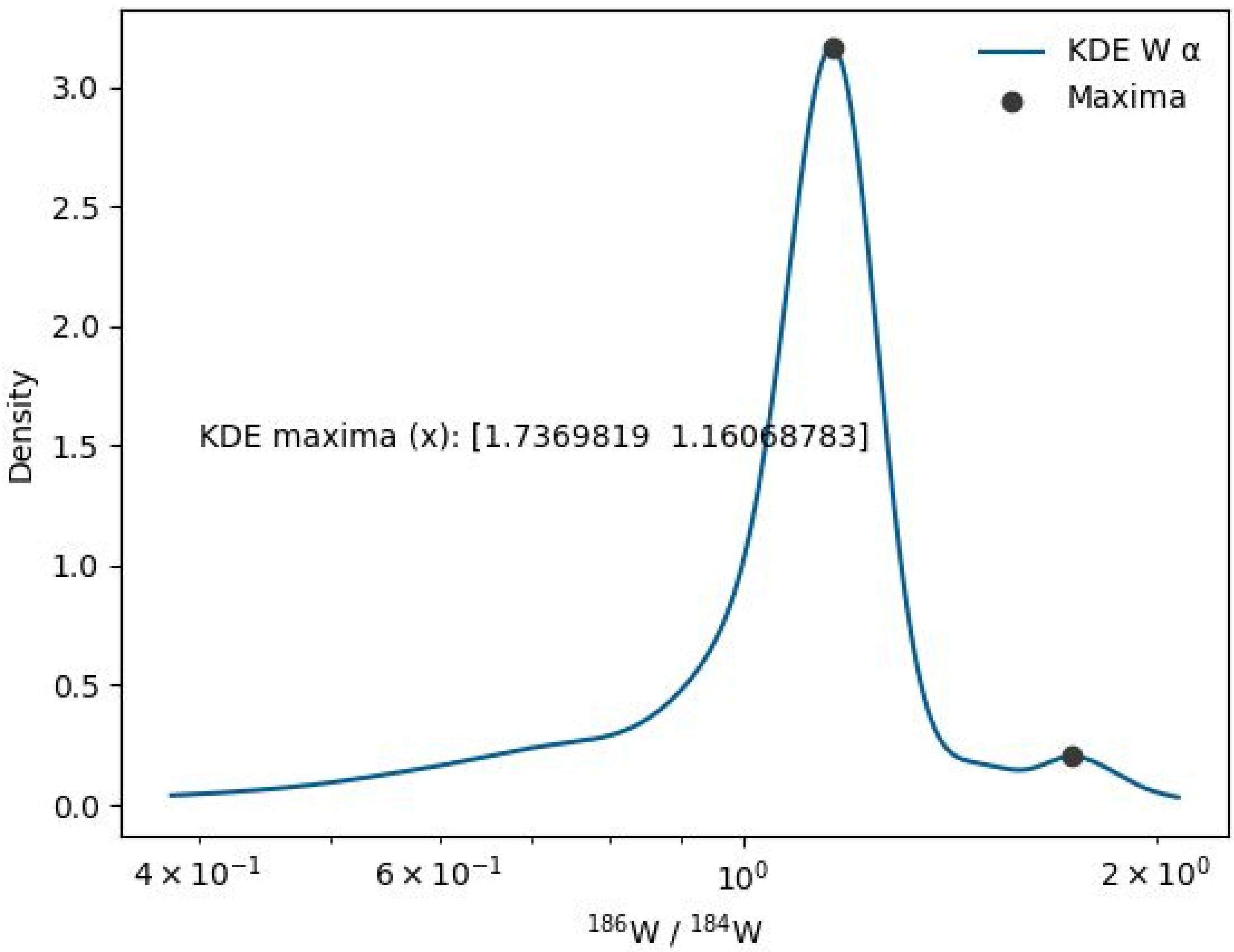

Figure S3. Replicates of Au analyses via SI-SP-ICP-MS.

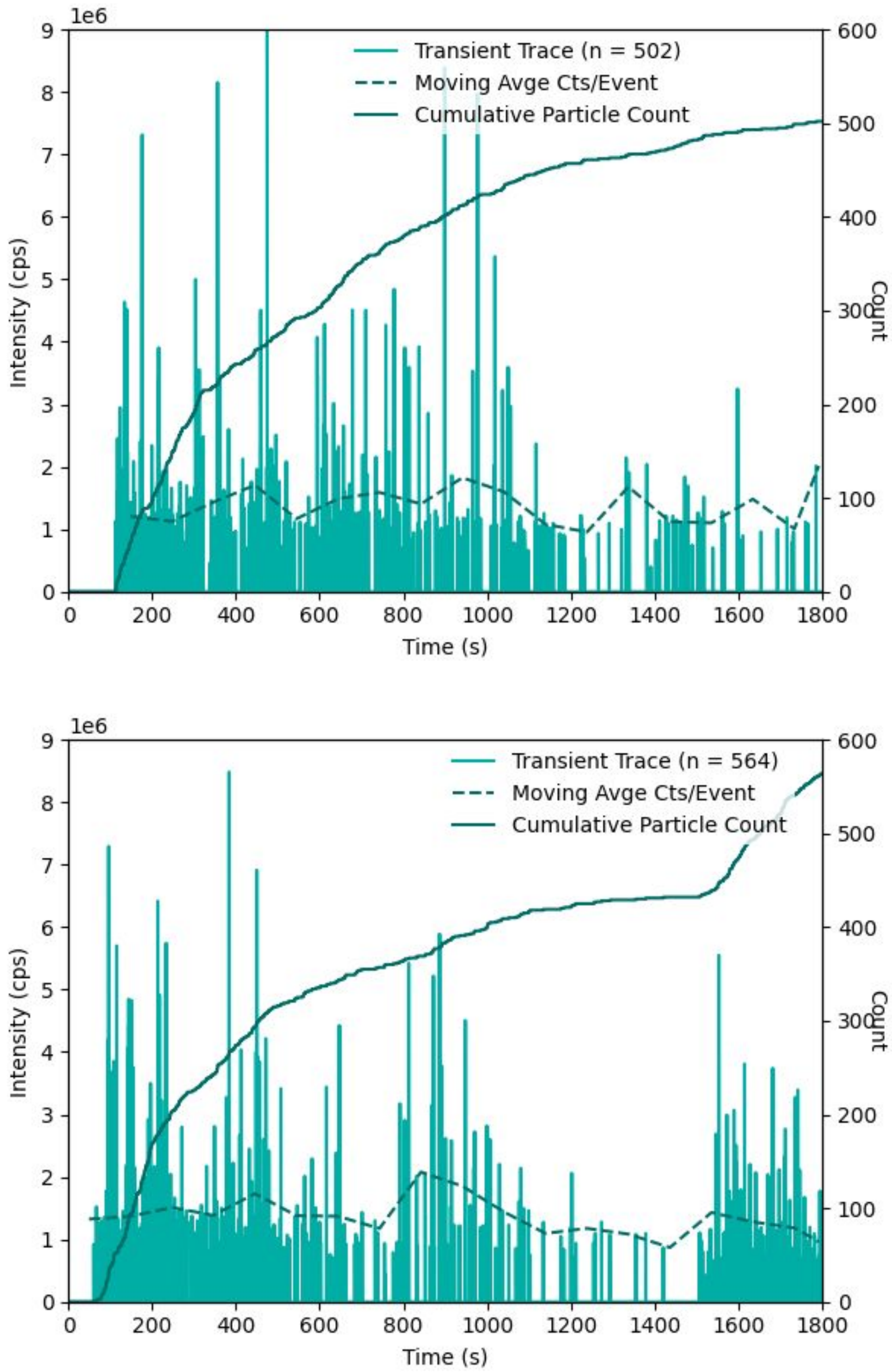

Figure S4. Time trace of Ni-α.

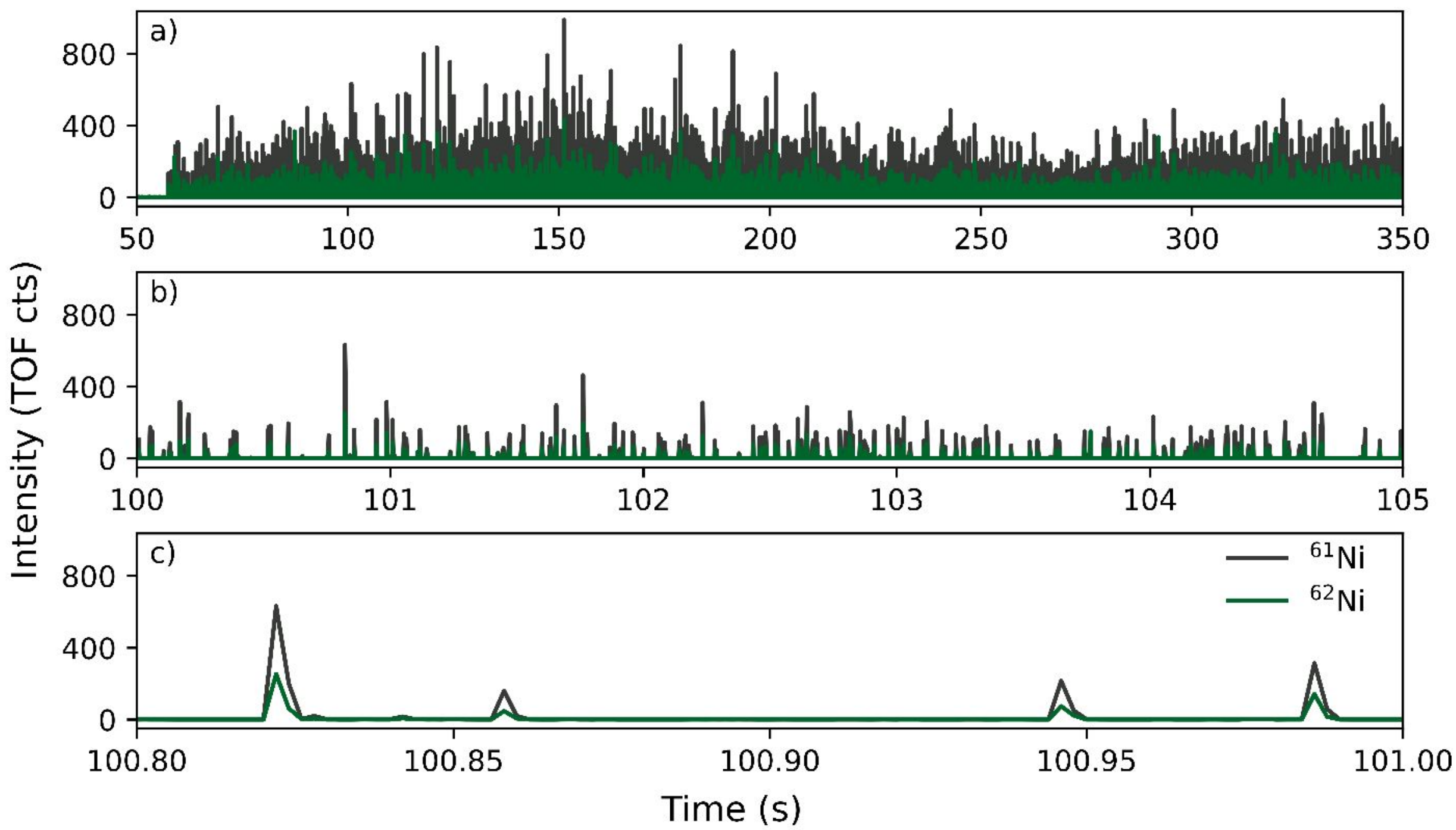

Figure S5. SEM images of Ni-α particles after analysis.

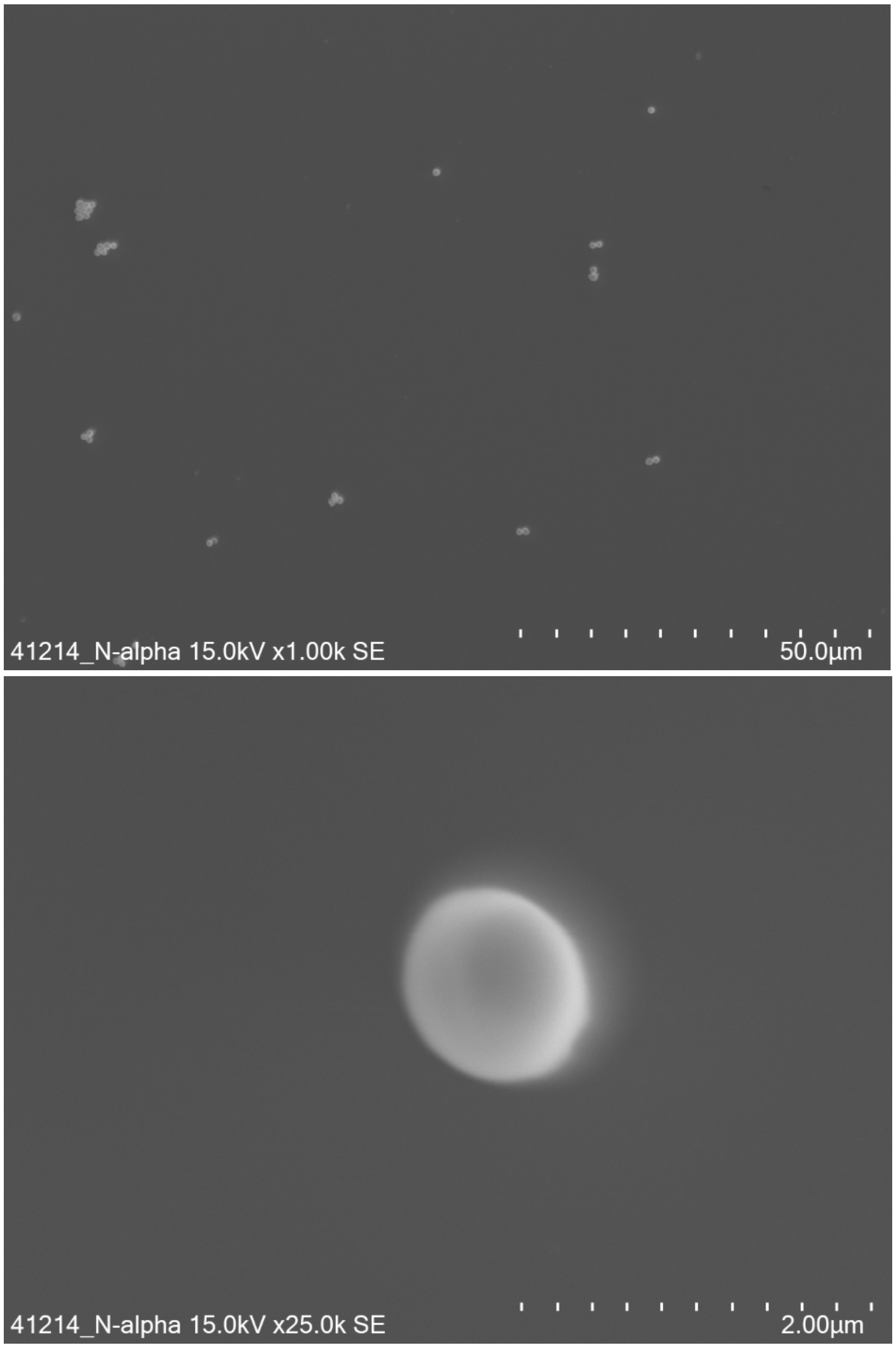

Equation S1. Determination of bandwidth for kernel density estimations of measured particle isotope ratios. n is the number of measurements and d is the number of spatial dimensions.

$$bandwidth = n^{\frac{-1}{d+4}}$$

Equation S2. Calculation of isotope ratios from delta values, where R stands for a given isotope ratio. See Scott (1992) and Virtanen et al. (2020).

$$R_{sample} = \left(\frac{\delta_{sample}}{1000} + 1\right) \cdot R_{standard}$$

Table S1. Heating of the sonicating cell during sonication.

**Sonication Duration (mins)**	**Interior Temperature (°C/°F)**	**Exterior Temperature (°C/°F)**
**0**	**21.4 / 70.5**	**21.4 / 70.5**
**5**	**26.8 / 80.2**	**26.1 / 78.9**
**10**	**27.0 / 80.6**	**26.8 / 80.2**
**15**	**27.1 / 80.7**	**26.8 / 80.2**
**20**	**27.2 / 80.9**	**27.0 / 80.6**
**25**	**27.6 / 81.6**	**27.4 / 81.3**
**30**	**27.5 / 81.5**	**27.6 / 81.6**

The temperature of the interior and exterior of the sonicating cell were measured using an infrared thermometer at a distance of ~3 inches.

References

D.W. Scott, “Multivariate Density Estimation: Theory, Practice, and Visualization”, John Wiley & Sons, New York, Chicester, 1992. https://doi.org/10.1002/9780470316849

Virtanen, P., Gommers, R., Oliphant, T.E. *et al.* SciPy 1.0: fundamental algorithms for scientific computing in Python. *Nat Methods* **17**, 261–272 (2020). https://doi.org/10.1038/s41592-019-0686-2
